# Communication Challenges During the Development and Introduction of a New Meningococcal Vaccine in Africa

**DOI:** 10.1093/cid/civ493

**Published:** 2015-11-09

**Authors:** Monique Berlier, Rodrigue Barry, John Shadid, Coimbra Sirica, Alison Brunier, Hayatee Hasan, Enricke Bouma

**Affiliations:** 1 Meningitis Vaccine Project, PATH, Ferney-Voltaire, France; 2 Inter-country Support Team for West Africa, World Health Organization, Ouagadougou, Burkina Faso; 3 United Nations Children's Fund, West and Central Africa Regional Office, Dakar, Senegal; 4 Burness Communications, Bethesda, Maryland; 5 Department of Communication Capacity Building; 6 Department of Immunization, Vaccines and Biologicals, World Health Organization, Geneva, Switzerland

**Keywords:** advocacy and communication, messaging, vaccine introduction, meningitis belt, crisis communication

## Abstract

***Background.*** A new group A meningococcal conjugate vaccine was developed to eliminate deadly meningitis epidemics in sub-Saharan Africa.

***Methods.*** From the outset of the project, advocacy and communication strategies were developed and adjusted as the project evolved in Europe, Africa, India, and the United States. Communications efforts were evidence-based, and involved partnerships with the media and various stakeholders including African ministries of health, the World Health Organization, UNICEF, Gavi, the Centers for Disease Control and Prevention, and Médecins Sans Frontières.

***Results.*** The implementation of an integrated communication strategy ensured the active cooperation of stakeholders while providing an organized and defined format for the dissemination of project-related developmental activities and the successful introduction of the vaccine.

***Conclusions.*** Early in the project, a communications strategy that engaged stakeholders and potential supporters was developed. The strategy was implemented and adapted as the project matured. Linked communication proved to be key to the successful wide-scale introduction of the PsA-TT (MenAfriVac) vaccine in Africa.

Meningitis epidemics have plagued sub-Saharan Africa for more than a century, causing immense human suffering for families and communities. In 2001, the Meningitis Vaccine Project (MVP)—a partnership between the World Health Organization (WHO) and PATH, with support from the Bill & Melinda Gates Foundation—was established with the mission of eliminating epidemic meningitis as a public health problem in sub-Saharan Africa through the development, testing, introduction, and widespread use of conjugate meningococcal vaccines. As a result of MVP, a new conjugate vaccine against group A *Neisseria meningitidis*, the PsA-TT vaccine (MenAfriVac), was developed and provided African health authorities, for the first time, with an affordable, long-term solution that protects against meningitis. Communications activities in support of MVP were essential and had to be strategically adapted to the development and introduction cycles of this long-awaited vaccine. This paper reviews the major strategic communications and advocacy initiatives that contributed to the success of MVP.

## EVOLUTION OF MVP COMMUNICATION STRATEGY

Initial steps focused on developing communication materials and a strategy targeting audiences in developing as well as in industrialized countries (decision makers, health officials, organizations and alliances, partners, and the private sector). The foundation of all activities was communication based on scientific evidence. A communications officer was hired at the project headquarters in Europe; a logo was designed; a website (www.meningvax.org) was created; an in-house scientific library was established; and a variety of fact sheets, question-and-answer documents, and brochures were developed in both French and English. Efforts were made to ensure consistent messaging within the WHO-PATH partnership that constituted MVP.

Close collaboration between researchers and the communications team ensured that peer-reviewed findings could be released under embargo, resulting in widespread media coverage of research confirming the impact of the vaccine in respected media worldwide.

As the project matured, the focus shifted to advocacy for stakeholders and potential supporters. The project team visited key meningitis belt countries to introduce the vaccine to healthcare authorities and to meet local journalists. It soon became clear that many local journalists would benefit from a better scientific grounding to help them understand the aim of the project. A better comprehension of the scientific and public health foundations of the project would enable them to provide science-based reports as the vaccine was introduced in Africa. To meet this need, regular media workshops in Africa were included in the communications plan.

## STAKEHOLDERS

A phase 1 clinical trial was conducted in India, the country where the manufacturer is based. After the completion of this phase 1 clinical trial [1], the MVP clinical activities shifted to Africa, as did the communication activities. In 2004, a Project Advisory Group was convened that consisted of African health, scientific, and communication experts [2].

An African communications officer with public health expertise was hired and based at the WHO Inter-country Support (IST) office in Ouagadougou, Burkina Faso. His role proved to be crucial in promoting and advocating for the vaccine's use while reporting on the project's progress from an African perspective. Over time, the European and African communications officers developed a comprehensive communications plan to complement the MVP operational work in target countries.

During decades of meningitis epidemics, African public health officials, families, and communities in the meningitis belt countries had become increasingly frustrated at not being able to better protect their populations against meningitis epidemics. Expectations were high that a new meningococcal vaccine could offer a real prospect of ending group A meningitis epidemics. Nonetheless, when MVP conducted its first field missions in Africa, the vaccine was still a distant and uncertain hope. Hence, promoting a product still in early development was a daunting communications challenge.

Setting up MVP's clinical trials in Africa (The Gambia, Mali, Senegal, and Ghana) entailed promoting the project with a series of activities, including country visits, workshops, targeted radio and television broadcasts, articles, regular updating of the MVP website, and production of quarterly electronic newsletters to keep stakeholders updated on the project's progress.

To enhance the scientific knowledge of journalists in clinical trial countries, MVP and partners organized a series of national workshops, inviting 20‒30 health communicators and journalists (from TV, radio, and press) to each workshop in Mali, The Gambia (both 2006), Senegal (2007), and Ghana (2008). The workshops offered an introductory course on why a meningitis vaccine clinical trial was taking place in their country, meningococcal disease and the epidemiology of meningitis, vaccine development, MVP, and vaccine introduction strategies. Whenever possible, the workshops included a visit to the clinical site so that journalists could tour the facilities and talk to the clinical team. Reporters were not expected to produce stories following the visit, but the tour was a way to foster a relationship between the clinical site and the media. On the final day of the workshop, participants wrote communication plans aimed at facilitating the conduct of the clinical trial in their country. However, as time went on it became apparent that lack of financial resources and qualified staff in media outlets made implementation of the proposed activities difficult.

Nevertheless, these workshops encouraged country ownership and the formation of communication networks that later became the foundation of the successful promotion of the vaccine when it was rolled out. All participants in the MVP workshops became part of the “MVP communication network” and received the quarterly newsletters, which regularly apprised them of the latest news on the development of the candidate vaccine, and later, on its rollout.

Although these workshops were not repeated for subsequent clinical trials in the same country, all clinical sites had access to “long-distance communication coaching” during the duration of the trials. Both the Europe- and Africa-based communicators provided this assistance, which covered areas such as writing communiqués for local use, developing visual aids for clinical staff and trial participants, and responding to rumors and political events that sometimes threatened the conduct of clinical studies. When needed, communication support was also provided to research partners. For example, when the London School of Hygiene and Tropical Medicine and the Centers for Disease Control and Prevention, Atlanta, initiated meningococcal carriage studies in Africa, communications plans were collaboratively developed to explain research activities that local populations might have considered intrusive (eg, collecting oropharyngeal swabs). To keep stakeholders informed about the trial and project progress, MVP also made targeted informational visits in each country where a trial was conducted (Table 1).

**Table 1.** Main Communication Stakeholder Targets (Primary, Secondary, and Tertiary)

Primary	Secondary	Tertiary
Ministers of health	Foundations	Scientists
Media	Governments	Ministries
Community leaders	Funding institutions	Community leaders
Target populations (ages 1–29)	Operators/private sector agents	Populations, communities
Regional institutions	Patrons	Regulatory authorities
Healthcare staff	Individuals	Ethics committees

## DEVELOPING KEY MESSAGES

Historically, reactive mass vaccination campaigns with polysaccharide vaccines that offered only short-term protection had been the norm in the meningitis belt, with these campaigns taking place whenever a meningitis epidemic occurred [3] The new PsA-TT conjugate vaccine, MenAfriVac, was designed to provide long-lasting protection and, as a result, to control group A meningitis epidemics in Africa. Messages related to the vaccine and the nature of future vaccination campaigns had to be adapted to the new product, and it was important to define and emphasize its comparative advantages. One salient supportive argument was that a similar vaccine—a conjugate vaccine against group C meningococci—had already proven its worth in the United Kingdom and several European countries.

With the success of the group C vaccine as a starting point, communication efforts concentrated on convincing stakeholders of the added value of using a conjugate vaccine instead of the polysaccharide vaccines that were already in use—namely, that the new vaccine was expected to protect younger children and to confer herd protection. Also, the possibility of someday introducing the vaccine as part of the Expanded Programme on Immunization routine schedule was cited as another major advantage, as well as the low price and the ability to use the vaccine preventively.

A significant challenge was how to present the need for the vaccine without making it appear to be an emergency. This was the critical and prime justification for the early media workshops. Attendees served as primary communications targets for the duration of the trials and were key in preventing the spread of misinformation. Similarly, researchers, academics, and local international press correspondents were also targeted. Keeping these stakeholders and players up-to-date from the beginning meant ensuring ownership while including them as key partners who could engender trust, argue for the project, and defend the project should local opposition arise. In the target countries, the media actions focused on basic information, presenting the project, and announcing the coming availability of an effective vaccine against the most fatal form of meningitis in the near future.

## POLITICAL CHAMPIONS FOR “ELIMINATING MENINGITIS EPIDEMICS IN AFRICA”

In 2006, the results of the first clinical studies confirmed the vaccine's major preventive potential, and a new initiative was launched to give maximum visibility to the vaccine and to the project. This effort included the appointment of a prominent African “patron” to lead peer-to-peer advocacy at the highest political levels with 2 aims: (1) to facilitate acceptance and introduction of the new vaccine; and (2) to help mobilize sufficient resources to vaccinate all at-risk populations.

The WHO Regional Office for Africa (AFRO) approached the President of Burkina Faso to serve as patron, as Burkina Faso had historically been the country most affected by group A meningitis epidemics. The Director-General of WHO had previously appointed him as an ambassador for the fight against neglected tropical diseases; thus, he had already lent his support to WHO AFRO to mobilize resources to fight epidemics. As chief advocate for the new PsA-TT vaccine, the President could make public declarations and provide high-level support during these crucial stages.

As MVP patron, the President held a series of meetings with Dr Luis Gomes Sambo, then Regional Director of WHO AFRO, during visits to Burkina Faso. Each of the meetings was an opportunity to communicate with the Burkina Faso–based national and international media.

The first notable achievement of the high-level patronage strategy occurred in September 2008, during the 58th session of the WHO Regional Committee when African ministers of health from meningitis belt countries unanimously adopted the Yaoundé Declaration, committing themselves to introduce “a highly promising candidate meningitis vaccine  …”. This public statement from the leadership of countries where the new vaccine would be introduced—made when the early MVP African clinical trials were still ongoing (PsA-TT-002 and PsA-TT-003), and before the first infant trial had begun (PsA-TT-004)—provided early crucial support for the aims of MVP [4, 5].

## POLITICAL WILL AND COMMITMENT

Another major step committing African leaders to the introduction of MenAfriVac took place on 24 June 2010, when Dr Sambo announced that the vaccine had been prequalified by WHO. This sent a clear signal to all meningitis belt countries that the vaccine met international standards of quality, safety, and efficacy and that its large-scale introduction in Africa could begin. It was a happy coincidence that Dr Sambo was on an official visit in Burkina Faso when the prequalification news broke; this high-level announcement was made at the end of Dr Sambo's presidential meeting and generated a strong signal of local political will to support MenAfriVac introduction in Africa.

Presidential support for the meningitis initiative culminated in the official MenAfriVac launch on 6 December 2010, in Ouagadougou, Burkina Faso's capital. Under the patronage of the President, the launch ceremony was held in the presence of key African political leaders and partners, including high-level representatives from PATH, WHO, Serum Institute of India, Ltd (the manufacturer of the vaccine), the Bill & Melinda Gates Foundation, the United Nations Children's Fund (UNICEF), and Gavi.

Communications preparations for the event had started in April, and by December, a package of information materials in French and English had been developed; partners had created special “MVP/MenAfriVac” sections on their organizational websites (including audiovisuals and stories from the field); desk-side meetings with science journalists had been held in London and Paris; communications teams from partnering organizations were deployed on the ground; filming crews were on standby to document the launch and the first vaccinations; and correspondents from African, European, and Asian media were on the ground in Burkina Faso to cover the event [6–8].

These activities—the largest deployment of public relations efforts since the creation of the project—were successful in raising global awareness about MenAfriVac and its potential to eliminate epidemic meningitis as a public health problem in Africa. News of the launch was covered over the next several weeks by major news agencies and media (print, radio, television) around the globe.

MenAfriVac had been the subject of news stories in the past, but coverage of the launch represented a sea change in the way journalists covered the vaccine. For the first time, media reported the new vaccine as a breakthrough—not just in combating meningitis epidemics, but as a new model to develop vaccines and drugs, particularly for developing nations, with MVP acting as a virtual vaccine development company that orchestrated the work of partners around the globe. The successful development and introduction of MenAfriVac in Africa was eventually recognized as one of the top health global success stories in 2010 [9–11].

## MESSAGING AND SOCIAL MOBILIZATION FOR THE VACCINATION CAMPAIGNS

### Adapted Messaging

It is worth noting that the key messages developed for global media outreach at the launch of the vaccine were different from those created by partnering institutions for social mobilization and to advocate for the vaccine's introduction in Africa. Whereas the launch messages emphasized the many “firsts” accomplished by MVP/MenAfriVac (first vaccine developed especially for Africa, introduced in Africa before being available anywhere else, first vaccine to be developed outside “big pharma,” vaccine developed at a remarkable initial cost of less than US$0.50 per dose), terms like “first/revolutionary/new/cheap” could have aroused suspicions in African populations; the terminology to be used was therefore left to the discretion of African stakeholders who could make that decision based on their own cultural environment.

### Young People: A Special Challenge

The target age group for the vaccine is 1- to 29-year-olds. From the first campaigns in 2010, it was clear that it was a challenge convincing adolescents and young adults—those between 15 and 29 years of age (especially males)—to take part in the vaccination campaigns.

MVP employed several new strategies, with the support of local and UNICEF specialists in social mobilization, that contributed to success in increasing vaccination rates in this population. These included peer education, vaccination lines for young boys only, targeted social mobilization messages, the participation of celebrities known by young people, and the launch of vaccination campaigns in universities and schools. In other populations, community discussions with the aim of engaging tribal and administrative leaders, or the use of social mobilization through public criers and other techniques, before, after, and especially during the official 10 days of the vaccination campaign, improved outreach to the desired targets. The same approaches and strategies, involving administrative, tribal, and religious leaders, were applied in Nigeria, a country that introduced the vaccine over 4 years. This led to an increase in participation among young people and a demand for additional vaccine doses in 2013 during the subsequent phase of introduction.

## ADVOCACY AND COMMUNICATION STRATEGY DURING ROLLOUT

### Workshops and Media Relations

Following introduction in Burkina Faso, Mali, and Niger, another dozen countries in Africa organized mass campaigns in several phases. In each country, communication workshops were jointly conducted by staff from the UNICEF West and Central Africa regional office in Dakar (WCARO) and WHO IST in Ouagadougou to inform communication and health professionals about MenAfriVac's introduction in the country. When deemed necessary, additional workshops were organized, focusing on the development of country-specific communication plans and messages.

Each workshop sought to boost the skills of the journalists to report on public health. Terms such as adverse events following immunization (AEFI) were explained, so that journalists could use them appropriately when communicating with the general public. They were therefore invited to participate in the technical committees set up to support the running of the vaccination campaigns where campaign results were discussed and explained. Finally, it is important to note that in 2012 the communication budget for the campaign was increased significantly, thanks to the financial support of the Gavi Alliance.

### Evaluation of Communication Efforts

During the MenAfriVac campaigns, according to information collected in a survey conducted by campaign supervisors in Burkina Faso, information on the vaccine and the campaign in that country came from a variety of sources. Social and media mobilizers accounted for 65% of the information sources, followed by healthcare agents (24%) and religious leaders (11%). If >12 million Burkinabé were vaccinated in 10 days, it was because they were duly informed. Indeed, 95% of respondents in the survey said they were aware of the campaign, and 98% were able to give the name of the disease they were being immunized against. The survey results suggest that communication efforts played a key role in the success of the vaccination campaigns.

### Capitalizing on Events: An Approach for Higher Visibility

The communications plan included identification of events that could maintain or renew media interest in the MenAfriVac Project in Africa or globally. Two such events included the symbolic celebration of the 100-millionth vaccination and the first distribution of MenAfriVac in a controlled temperature chain (CTC) [12, 13].

According to data gathered in the first 10 countries to introduce MenAfriVac, some 103 million people had been vaccinated by the end of 2012. This was of particular importance in Africa, and proactive communication raised the visibility of this milestone. The choice of Benin as the country where the celebration would be held was not only practical—the country was organizing a campaign in 2012—but above all, it was a geostrategic and political choice because the Benin President was also President of the African Union. He would be in an excellent position to promote the vaccine to other African heads of state where MenAfriVac was due to be introduced. The 15 November ceremony was broadcast live throughout Africa. The event led to several news stories in African media on the success of the MenAfriVac campaigns. Not only was a symbolic milestone acknowledged, but awareness of the risks of meningitis was raised for countries where knowledge was more limited and introducing the vaccine might be more challenging.

As the Africa-based MVP media outreach from Cotonou catered to populations living in meningitis belt countries and African leaders, the European team took advantage of new findings on the stability of the MenAfriVac vaccine to reach out to the international scientific/vaccine community with news that MenAfriVac had become the first vaccine to receive regulatory approval to travel at up to 40° for up to 4 days in Africa in a CTC, thus breaking with the traditional mold of the 2‒8°C cold chain, which requires constant refrigeration. There was a risk that the new method could be viewed as dangerous or as “another vaccine test” conducted on African populations, which is why a strategic choice was made to first establish awareness in the scientific community. The 14 November CTC announcement—deliberately coincident with the Cotonou event—was made from the American Society of Tropical Hygiene and Medicine conference in Atlanta, Georgia [12], and attracted global attention because the new flexibility allowed by MenAfriVac's thermostability data could potentially be replicated for other vaccines.

Meanwhile, the announcement on the launch of the CTC pilot study in Benin provided an opportunity to follow up in February 2014 with a related paper on the economic benefits of vaccine distribution in CTC [13, 14]. The positive results from the first CTC vaccination campaign ever conducted in Africa were communicated in a targeted outreach to media and stakeholders in Africa and in other developing countries that might benefit from the new distribution method. The evidence showed that the CTC approach worked, that it was safe, and that it saved time, energy, and money; MVP communicators were confident that the news would be well received in regions where CTC had the potential to revolutionize vaccine distribution [13].

## WHEN THINGS GO WRONG: CRISIS MANAGEMENT TRAINING

Throughout the project's lifespan, training in crisis communications was a key component of the MVP communications strategy. For the clinical trials, this involved drawing up crisis communications plans to include all relevant partners, and a short training exercise to ensure that principal investigators and staff would know how to handle media queries and respond to potential crisis situations that might derail a clinical trial. Needless to say, community acceptance and an awareness of local sensitivities were crucial [15]

On a larger scale, crisis communication plans were drawn up and training was provided in preparation for the mass vaccination campaigns. In August 2010, the first crisis communication workshop was held to prepare for vaccine introduction. Jointly organized by PATH, WHO IST/West Africa, and UNICEF WCARO, managers and decision makers from health ministries along with communication officers in Burkina Faso, Niger, and Mali (the early adopters) were trained on the important elements in identifying and managing a crisis. This training included how to identify key technical personnel, interested politicians, and spokespersons and how to prepare a crisis management plan before a crisis occurs. The workshops were repeated for each new introducing country, either as national or multicountry workshops.

## CONTRASTING EXPERIENCES IN MANAGING A CRISIS

### Managing a Local Crisis in Burkina Faso

In December 2010, a crisis developed in Burkina Faso with the death of a child on day 8 of the planned 10-day vaccination campaign. News of the death was published on the front page of a national daily newspaper that linked the child's death to the vaccination (day 1 of the crisis). Following the advice of the crisis management team, consisting of both technical and communications experts, a task force was immediately sent out to investigate and a response prepared. The following day a press conference was held, led by the Minister of Health, surrounded by his staff and key partners (WHO and UNICEF), that described the results of the investigation. The press conference was held sufficiently early in the day to enable declarations from the national healthcare authorities to be written and published the following day (day 3) and report that the death of this child was due to a prior illness and unrelated to the vaccine. This quickly put an end to articles that questioned the quality of the vaccine.

Special attention was devoted to the social and cultural management of the child's death. A local mission was assembled, consisting of tribal, administrative, and elected representatives, to offer condolences to the grieving family. Employing these parallel approaches defused the Burkina Faso crisis, which had the potential to derail not only the campaign in Burkina Faso, but also the introduction of MenAfriVac in other countries. The timely and professional handling of the Burkina Faso crisis at local and national levels enabled the continuation of the mass vaccination campaign with no impact on vaccine introduction in other countries.

### Mass Psychogenic Illness Following MenAfriVac Introduction in Gouro, Chad

Nothing in the successful management of the Burkina Faso crisis or the smooth introduction of MenAfriVac in 7 countries in 2011‒2012 foreshadowed the crisis that snowballed from Gouro, an isolated settlement in northern Chad, into a wave of anti-MenAfriVac stories worldwide in early 2013. Chad had successfully conducted 3 vaccination rounds with MenAfriVac in other parts of the country in 2011 and 2012 that immunized 7.2 million Chadians with no safety concerns. The Ennedi region, where Gouro is located, was part of the fourth and final vaccination phase in Chad. The campaign, which had started in Gouro on 11 December 2012, was abruptly stopped on December 15 when vaccinated children reportedly fell ill.

The incident prompted a Chadian journalist (subsequently identified as a regime opponent) to post a story on December 22 that described 40 children who had become severely ill (some with paralysis) in Gouro after being vaccinated with MenAfriVac. Within 24 hours the story was reposted by an online national news channel, and a few days later the information made headlines in Chadian print and online media. The story was subsequently picked up by European and US antivaccination activists, who accused MVP and partners of deliberately committing genocide among Africa's poorest and most fragile populations. The story was published on the website of an international news agency 41 days after the publication of the first blog, but was removed within a couple of hours thanks to the swift intervention of MVP communicators.

Chadian authorities invited an international team of clinicians and epidemiologists to investigate the problem. An investigation proceeded; all cases were examined by physicians, who did not find cases of paralysis, and all of the affected individuals recovered without incident. These findings were published in an official report. The episode was determined to be “mass psychogenic illness”—an unusual phenomenon that has been well described, the affected tending to be clusters of young girls with unusual clinical findings that gradually improve [16, 17]. Episodes have been related to vaccination campaigns done during times of political instability and general mistrust, as was the case in Chad in 2013. The political backdrop helps explain the slow reaction by health authorities. Suspecting political mischief, members of the government chose to address the situation privately or not to react at all, giving free rein to rumors and exaggeration, especially in the written media, which led to a worsening crisis at the local, national, and international levels.

In the end, the daily visits of expert clinicians to hospitalized children in N'djamena, the accessibility of the authorities to local leaders, and discussions with the ministry of health brought a satisfactory outcome to this crisis. It ultimately created an opportunity to communicate positive messages on the vaccine, recognizing it as safe and of good quality. Bearing the signature of the health minister, 2 communiqués were distributed to break the “radio silence” and get back in touch with the general public, who were asking for official information. These communiqués, one of which reported on the result of the investigation published on 21 January, were used by most of the media, helping to calm the tense social climate [18, 19]. Nonetheless, the Gouro episode was a sobering example of the power of rumor and communication, and of the necessity of developing and implementing comprehensive crisis communication plans.

## LESSONS AND LEGACY AFTER IMPLEMENTATION OF THE MVP PROJECT

The MenAfriVac mass vaccination campaigns in Africa will go down in history as a stunning success. One year after large-scale vaccine introduction in late 2010, experience from Burkina Faso provided early evidence that mass vaccination was associated with a significantly reduced risk of meningitis in the targeted population, as well as among the unvaccinated age groups, suggesting that MenAfriVac induced “community” protection. Findings were confirmed in a major way in Chad in 2012 where researchers reported a dramatic reduction in transmission and incidence of group A meningococcal disease, a drop of >90% following vaccination [20].

Some lessons are worthy of note for future communication campaigns. Key factors for success included good planning, with suitable strategies and an approach that built to a crescendo, the engagement of stakeholders and potential supporters, and timely allocation of resources. Effectively promoting MVP with well-defined targets and objectives was accomplished by pooling skills and expertise, both within the communications team and with partners holding distinct, but complementary, responsibilities.

The training courses proved to be a bonus that will be part of MVP's legacy. Although many countries were doubtful about the need for crisis communication training, the postworkshop feedback clearly indicated that the participants considered them to be valuable. To be most effective, this type of training should be an integral part of vaccine introduction. The “crisis” experiences in Burkina Faso and in Chad raised awareness of the importance of communications, which, if not managed, can have dramatic repercussions.

In addition, we note 3 important factors that have to be taken into account by all players if communication efforts are to succeed:

- An advocacy and communication strategy that is woven into the fabric of the project and that evolves dynamically with all aspects of the project;
- A culturally appropriate approach;
- Boosting of the skills of healthcare teams in community dialogue and other interactive techniques if necessary.

The implementation of MVP enjoyed a significant advantage: It had an excellent vaccine protecting people against a killer disease. But it is worth remembering that even fear does not always spur action, and often action does not last. One needs correct strategic communication choices to instill lasting trust within communities.
